# Statins Inhibit Inflammatory Cytokine Production by Macrophages and Acinar-to-Ductal Metaplasia of Pancreatic Cells

**DOI:** 10.1016/j.gastha.2022.04.012

**Published:** 2022-04-25

**Authors:** Soichiro Ako, Yaroslav Teper, Linda Ye, James Sinnett-Smith, Oscar J. Hines, Enrique Rozengurt, Guido Eibl

**Affiliations:** 1 Department of Surgery, David Geffen School of Medicine, University of California, Los Angeles, California;; 2 Department of Medicine, David Geffen School of Medicine, University of California, Los Angeles, California

**Keywords:** Pancreatic Cancer, Statins, Macrophages, Acinar-to-Ductal Metaplasia

## Abstract

**BACKGROUND AND AIMS:**

Animal data show that the presence of an oncogenic Kras mutation in pancreatic acinar cells leads to acinar-to-ductal metaplasia (ADM), pancreatic intraepithelial neoplasia (PanIN), and pancreatic ductal adenocarcinoma (PDAC). Inflammatory macrophages play an important role in the formation of ADMs and transition to PanINs. Epidemiologically, statins are associated with a reduced risk of PDAC. We investigated whether statins inhibit inflammatory cytokine production in macrophages and whether this leads to reduced ADM formation.

**METHODS:**

The efficacy of statins on inflammatory cytokine production in 2 macrophage cell lines was measured by real-time polymerase chain reaction and enzyme-linked immunosorbent assay. The effect of macrophage-conditioned medium on ADM in primary pancreatic acinar cells was investigated. Mouse pancreatic tissue samples were analyzed for macrophage numbers, cytokine levels, and neoplastic/dysplastic area.

**RESULTS:**

Lipophilic statins prevented inflammatory cytokine production in Raw264.7 and J774A.1 cells stimulated by lipopolysaccharide. The inhibitory effect of statins was mediated by inhibition of mevalonate and geranylgeranyl pyrophosphate synthesis and disruption of the actin cytoskeleton but not by a reduction in intracellular cholesterol. Treatment of macrophages with lipophilic statins also blocked ADM formation of primary pancreatic acinar cells. Furthermore, oral administration of simvastatin was associated with a reduction in the number of intrapancreatic macrophages, decreased inflammatory cytokine levels in the pancreas, and attenuated ADM/PanIN formation in mice.

**CONCLUSION:**

Our data support the hypothesis that statins oppose early PDAC development by their effects on macrophages and ADM formation. The inhibitory actions of statins on macrophages may collaborate with direct inhibitory effects on transformed pancreatic epithelial cells, which cumulatively may reduce early PDAC development and progression.

## Introduction

Pancreatic ductal adenocarcinoma (PDAC) remains one of the most deadly malignancies with an overall 5-year survival of around 9%.^1^ Even though substantial progress has been made in surgical outcomes of a small proportion of patients with resectable disease as well as in understanding driving genetic alterations and oncogenic signaling networks, targeted molecular therapy offers only a marginal survival benefit. Increasing efforts should be directed toward attempts to prevent PDAC or halt the progression of precursor lesions to invasive disease (interception).^2^ This is particularly important as the incidence and mortality of PDAC is expected to rise,^3^ which may be caused largely or at least partially due to the high and rising prevalence of obesity in US adults and children.^4^

Acinar-to-ductal metaplasia (ADM) in the pancreas is often seen as a response to pancreatic inflammation, for example, during pancreatitis, but is usually reversible once the triggering stressor subsides.^5,6^ However, in the additional presence of an oncogenic *Kras* in pancreatic acinar cells, ADMs persist and may progress to pancreatic intraepithelial neoplasias (PanINs),^6^ recognized neoplastic precursor lesions of PDAC. Experimental animal studies have shown that proinflammatory M1 macrophages are instrumental and indispensable for ADM formation.^7,8^ In this context, pancreatic acinar cells that express oncogenic Kras attract M1 macrophages to induce local inflammation. Once in the pancreatic microenvironment, M1 macrophages accelerate acinar cell trans-differentiation through the secretion of proinflammatory cytokines. Depletion or inhibition of macrophages by different approaches has been shown to prevent pancreatic inflammation and formation of ADM and subsequent PanIN lesions.^8^ In contrast, in more advanced stages of PDAC development, that is, PanIN-3 and invasive cancers, macrophages exhibit generally immune-suppressive, and hence protumorigenic, properties.^9^

Several epidemiologic studies have shown that statins, Food and Drug Administration-approved drugs to treat hypercholesterolemia, are associated with a reduced risk of PDAC,^10–13^ a notion that is supported by preclinical genetically engineered animal studies.^14–17^ We have previously reported that diet-induced obesity (DIO) was associated with enhanced pancreatic inflammation and PDAC development in the *KrasG12D;p48-Cre* (KC) mouse model.^18,19^ In a subsequent study, we demonstrated that statins attenuated pancreatic inflammation and PanIN progression in KC mice.^20^ Importantly, statins greatly reversed the pancreatic acinar cell loss seen in the pancreas of obese KC mice, suggesting an inhibitory effect on ADM formation. It is conceivable that statins elicit their beneficial effects on PDAC development by direct effects on transformed pancreatic epithelial cells, as shown by us previously,^20^ and by inhibiting inflammatory cytokine production by macrophages resulting in reduced ADM formation.

We now report that statins inhibit proinflammatory cytokine production in murine macrophages in cell culture models through inhibition of the mevalonate pathway, reduction of downstream geranylgeranyl-pyrophosphate (GG-PP), and disruption of actin cytoskeleton. This was associated with a reduction in intrapancreatic macrophages and cytokine levels, which correlated to a decrease in metaplastic/neoplastic lesions in the pancreas of KC mice treated with statins, and inhibition of ADM formation of primary pancreatic acinar cells.

## Results

### Lipophilic Statins Inhibit Lipopolysaccharide-Induced Production of Interleukin-6 in Macrophages

**Figure 1.**
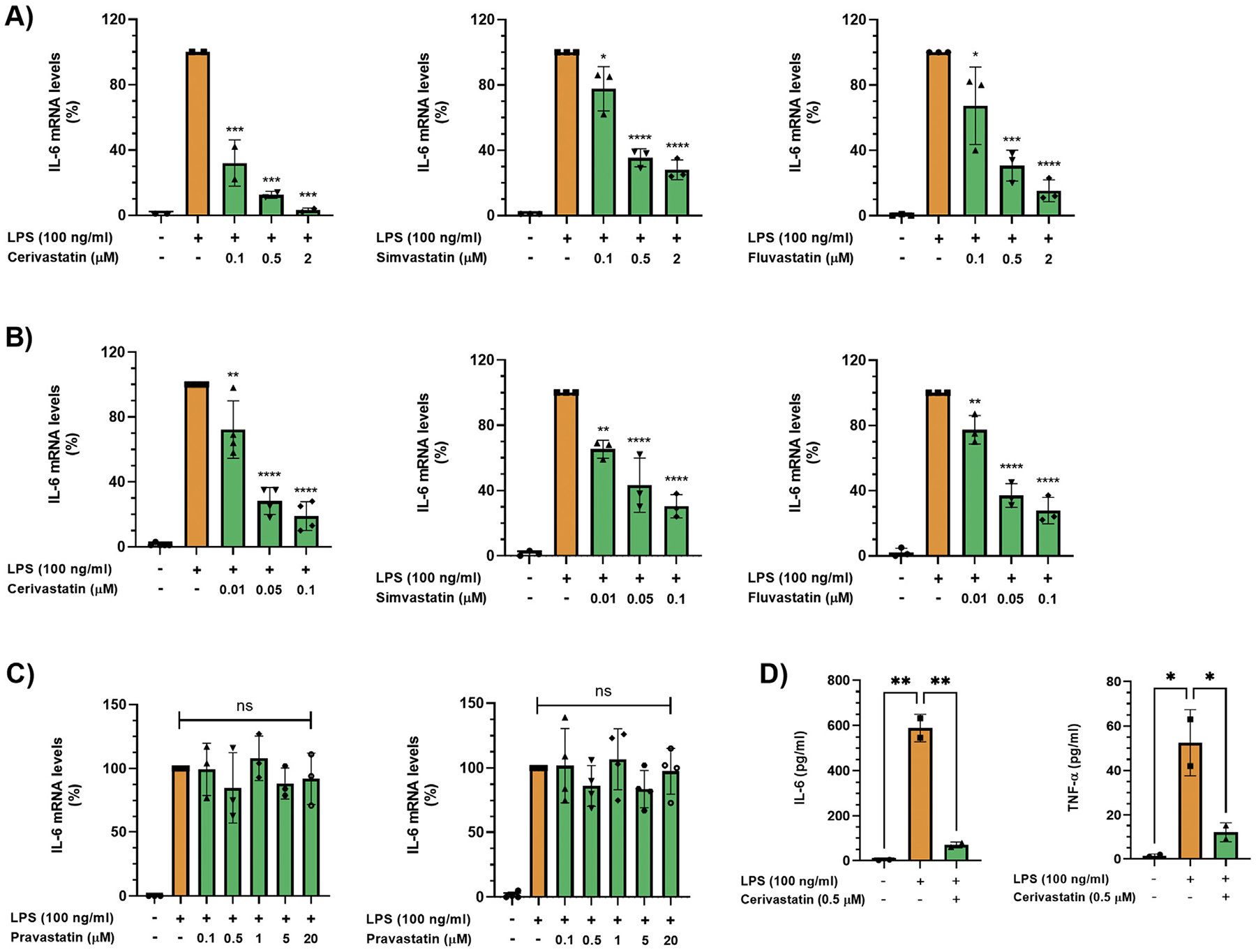
(A, B) Cells were pretreated with statins overnight in serum-free medium. Then, Raw264.7 (A) and J774A.1 (B) cells were treated with 100-ng/mL LPS in the absence or presence of different concentrations of cerivastatin, simvastatin, and fluvastatin for 6 hours. IL-6 mRNA levels were measured by real-time qPCR. Real-time PCR data were standardized to cells treated with LPS only (set to 100%). Data are presented as scatter plots with bars (mean ± SD). Significances indicate comparison to cells treated with LPS only. *P ≤ .05; **P ≤ .01; ***P ≤ .001; ****P ≤ .0001. (C) Cells were pretreated with pravastatin overnight in serum-free medium. Then, Raw264.7 (left panel) and J774A.1 (right panel) cells were treated with 100 ng/mL LPS in the absence or presence of different concentrations of pravastatin for 6 hours. IL-6 mRNA levels were measured by real-time qPCR. Real-time PCR data were standardized to cells treated with LPS only (set to 100%). Data are presented as scatter plots with bars (mean ± SD). ns = not significant. (D) Raw264.7 cells were pretreated with cerivastatin overnight in serum-free medium. Then, cells were treated with 100-ng/mL LPS in the absence or presence of 0.5-*μ*M cerivastatin for 24 hours, and IL-6 (left panel) and TNF-*α* (right panel) protein levels were measured by ELISA. Data are presented as scatter plots with bars (mean ± SD). *P ≤ .05; **P ≤ .01. ELISA, enzyme-linked immunosorbent assay; qPCR, quantitative polymerase chain reaction.

To determine whether statins inhibit proinflammatory cytokine production in macrophages, we used the murine macrophage/monocyte cell lines Raw264.7 and J774A.1. Initially, Raw264.7 macrophages were exposed to different concentrations of lipopolysaccharide (LPS) (10, 50, 100 ng/mL) for 3, 6, or 24 hours and interleukin-6 (IL-6) and tumor necrosis factor-*α* (TNF-*α*) mRNAs were measured by real-time polymerase chain reaction (PCR). LPS dose-dependently increased IL-6 and TNF-*α* mRNA levels reaching peak values at 6 hours at all concentrations tested (Figure A1). Crucially, treatment of either Raw264.7 or J774A.1 cells with the lipophilic statins cerivastatin, simvastatin, and fluvastatin dose-dependently (0–2 *μ*M) attenuated the increase in IL-6 transcript levels induced by LPS (Figure 1A and B). In Raw264.7 cells, IL-6 mRNA levels were reduced to 12 ± 2%, 35 ± 5%, and 31 ± 9% with 0.5-*μ*m cerivastatin, simvastatin, and fluvastatin, respectively (Figure 1A). In J774A.1 cells, cerivastatin, simvastatin, and fluvastatin at 0.1 *μ*M reduced IL-6 mRNA levels to 19 ± 9%, 30 ± 7%, and 27 ± 8%, respectively (Figure 1B). In sharp contrast to the lipophilic statins, the hydrophilic pravastatin did not reduce LPS-stimulated IL-6 production in both cell lines, even at 20 *μ*M, the highest concentration tested (Figure 1C). Furthermore, exposure of Raw264.7 macrophages to 100-ng/mL LPS for 6 hours increased IL-6 and TNF-*α* protein levels, which was inhibited by 0.5-*μ*M cerivastatin (Figure 1D). In addition, using the Mouse Innate and Adaptive Immune Reponses RT2 Profiler Kit PCR Array containing primer assays for 84 genes, we found that cerivastatin (0.5 *μ*M) decreased the expression of multiple genes, including IL-6, TNF-*α*, IL-1*α*, IL-1*β*, and CCL5/Rantes in Raw264.7 cells exposed to LPS (100 ng/mL) for 6 hours (Table A1). Together, these data demonstrate that lipophilic statins prevent LPS-induced proinflammatory cytokine production in macrophages.

### Statins Inhibit LPS-Induced IL-6 Production in Macrophages by Inhibiting the Mevalonate but not the Squalene-Cholesterol Biosynthesis Pathway

**Figure 2.**
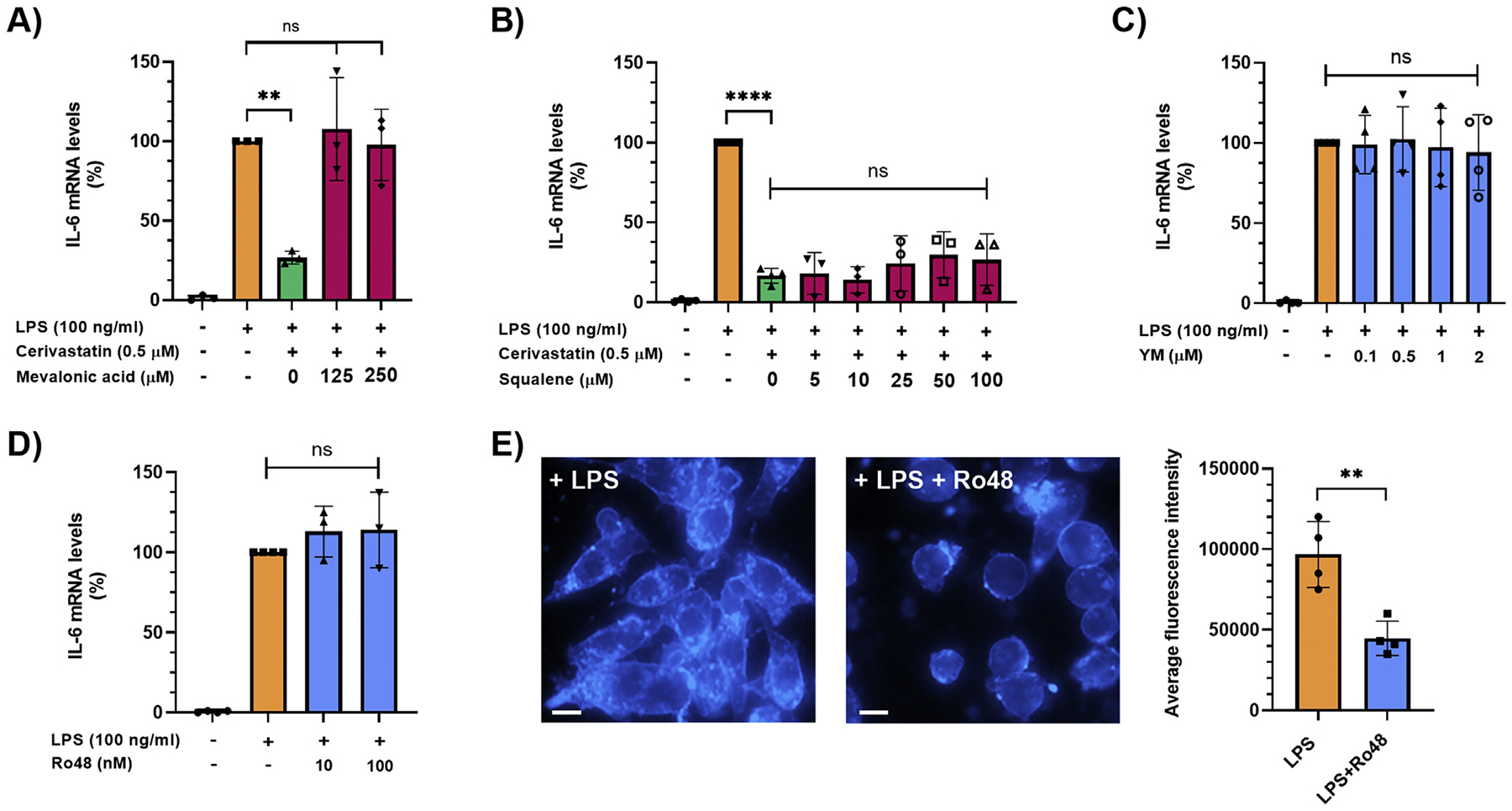
(A, B) Raw264.7 cells were pretreated with statins overnight in serum-free medium. Then, cells were treated with 100-ng/mL LPS in the absence or presence of 0.5-*μ*M cerivastatin and indicated concentrations of mevalonic acid (A) or squalene (B) for 6 hours. IL-6 mRNA levels were measured by real-time qPCR. Real-time PCR data were standardized to cells treated with LPS only (set to 100%). Data are presented as scatter plots with bars (mean ± SD). **P ≤ .01; ****P ≤ .0001; ns = not significant. (C, D) Raw264.7 cells were pretreated with inhibitors overnight in serum-free medium. Then, cells were treated with 100-ng/mL LPS in the absence or presence of indicated concentrations of the squalene synthase inhibitor YM-53601 (YM) (C) or the oxidosqualene cyclase inhibitor Ro 48-8071 (Ro48) (D) for 6 hours. IL-6 mRNA levels were measured by real-time qPCR. Real-time PCR data were standardized to cells treated with LPS only (set to 100%). Data are presented as scatter plots with bars (mean ± SD). ns = not significant. (E) Raw264.7 cells were pretreated with inhibitors overnight in serum-free medium. Then, cells were treated with 100-ng/mL LPS in the absence or presence of the oxidosqualene cyclase inhibitor Ro 48-8071 (Ro48) at 10 nM for 6 hours and stained with filipin III using the Cholesterol Cell-Based Detection Assay Kit. Representative images are shown. Scale bar, 50 *μ*m. Fluorescence intensity indicating intracellular cholesterol levels was quantified in 4 nonoverlapping areas with at least 20 cells per area using the Image J software (right panel). Average fluorescence intensity is presented as scatter plots with bars (mean ± SD). **P ≤ .01. qPCR, quantitative polymerase chain reaction.

Having shown that statins decrease the expression of multiple genes in LPS-stimulated Raw264.7 cells, we selected IL-6, which has been suggested to play an important role in ADM formation,^21^ for subsequent studies to dissect the underlying mechanisms. Statins are known inhibitors of 3-hydroxy-3-methyl-glutaryl-coenzyme A reductase, the rate-limiting enzyme of the mevalonate pathway, which produces cholesterol and other isoprenoids.^22^ To investigate the mechanism(s) by which statins inhibit LPS-induced increase of IL-6 expression in macrophages, Raw264.7 cells were exposed to 100-ng/mL LPS and 0.5-*μ*M cerivastatin for 6 hours either in the absence or presence of mevalonate (125 or 250 *μ*M). As seen in Figure 2A, exogenous mevalonate completely reversed the inhibitory effects of cerivastatin on LPS-induced IL-6 expression. In contrast, exogenous squalene, an intermediate in cholesterol biosynthesis generated by squalene synthase, had no significant effect on reversing the effect of cerivastatin (Figure 2B). Accordingly, YM-53601 (0–2 *μ*M), a squalene synthase inhibitor, also failed to inhibit the LPS-induced increase in IL-6 transcript levels in Raw264.7 cells (Figure 2C). To confirm the notion that the reduction of intracellular cholesterol biosynthesis by statins does not play a role in the inhibition of IL-6 expression by statins in macrophages stimulated with LPS, we exposed Raw264.7 cells to 100-ng/mL LPS together with the oxidosqualene cyclase inhibitor Ro 48-8071 (0–100 nM), a potent inhibitor of cholesterol biosynthesis. As shown in Figure 2D, Ro 48-8071 at 100 nM did not prevent LPS-induced increase in IL-6 transcript levels in Raw264.7 cells, while it significantly suppressed (by ~50%) intracellular cholesterol levels at this concentration, as detected by filipin immunofluorescence staining (Figure 2E). These data indicate that the inhibition of LPS-induced IL-6 production in macrophages by statins is mediated by inhibition of mevalonate synthesis but not by a reduction of intracellular cholesterol.

### Statins Inhibit LPS-Induced IL-6 Production in Macrophages by Inhibiting the Protein Prenylation Pathway and Disruption of the Actin Cytoskeleton

**Figure 3.**
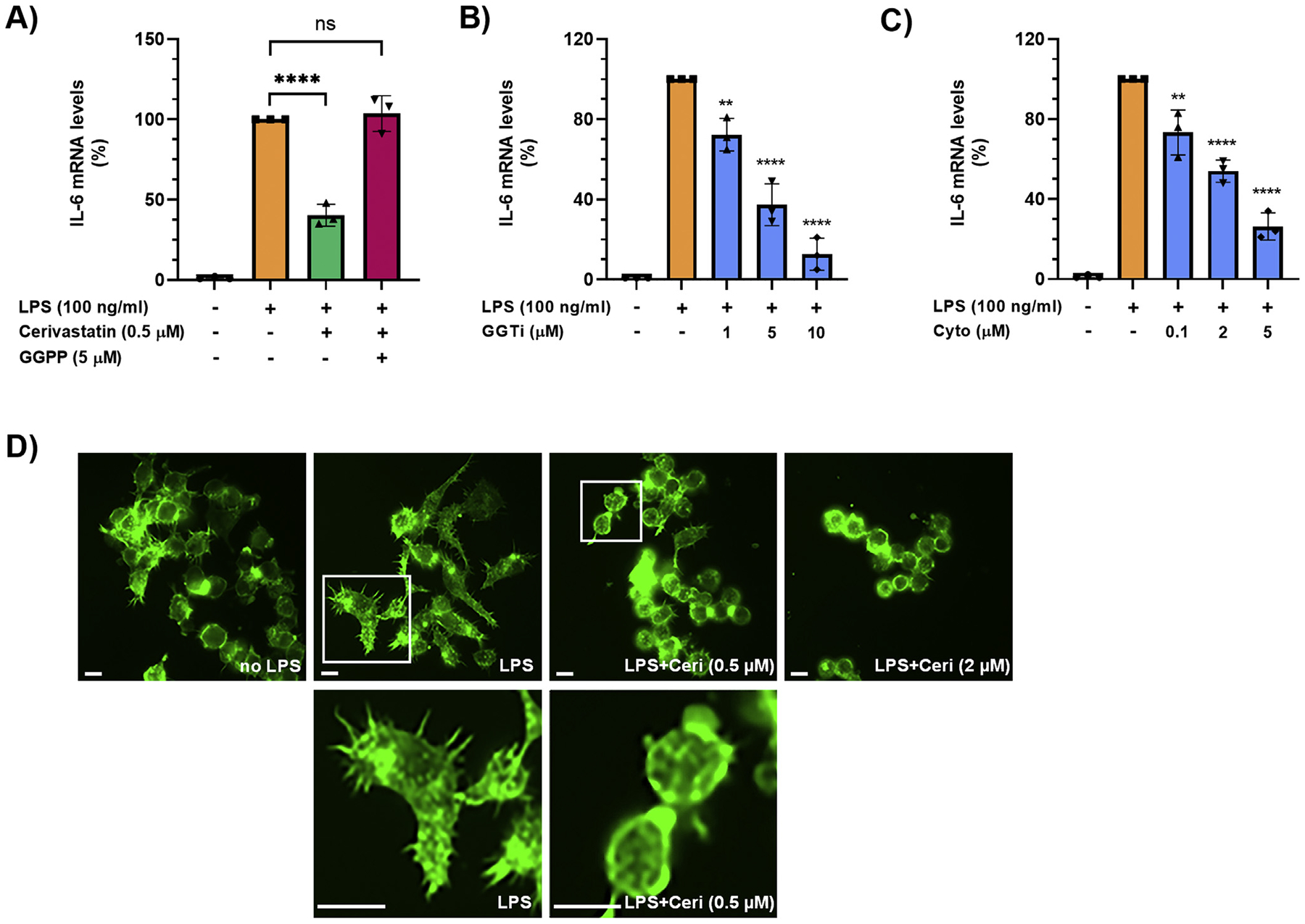
(A) Raw264.7 cells were pretreated with statins overnight in serum-free medium. Then, cells were treated with 100-ng/mL LPS in the absence or presence of 0.5-*μ*M cerivastatin and 5-*μ*M geranylgeranyl pyrophosphate (GGPP) for 6 hours. IL-6 mRNA levels were measured by real-time qPCR. Real-time PCR data were standardized to cells treated with LPS only (set to 100%). Data are presented as scatter plots with bars (mean ± SD). ****P ≤ .0001; ns = not significant. (B, C) Raw264.7 cells were pretreated with inhibitors overnight in serum-free medium. Then, cells were treated with 100-ng/mL LPS in the absence or presence of indicated concentrations of the geranylgeranyl transferase inhibitor GGTI 298 (GGTi) (B) or the actin polymerization inhibitor Cytochalasin D (Cyto) (C) for 6 hours. IL-6 mRNA levels were measured by real-time qPCR. Real-time PCR data were standardized to cells treated with LPS only (set to 100%). Data are presented as scatter plots with bars (mean ± SD). Significances indicate comparison to cells treated with LPS only. **P ≤ .01; ****P ≤ .0001.(D) Raw264.7 cells were pretreated with statins overnight in serum-free medium. Then, cells were treated without (no LPS) or with 100-ng/mL LPS in the absence or presence of 0.5-*μ*M or 2-*μ*M cerivastatin (Ceri) for 6 hours and then stained with Alexa Fluor 488 Phalloidin. Representative images are shown (upper row). Digitally zoomed-in areas are shown for cells treated with LPS or LPS plus 0.5-*μ*M cerivastatin to clearly show cell shape and filipodia. Scale bar, 50 *μ*m. qPCR, quantitative polymerase chain reaction.

Besides leading to cholesterol biosynthesis, the mevalonate pathway also produces farnesyl-pyrophosphate and GG-PP, important for protein prenylation, a post-translational lipid modification of proteins (eg, Rho family GTPases) that involves the addition of prenyl groups, for example, farnesyl or geranylgeranyl, thereby facilitating the attachment to cell membranes.^23^ In contrast to squalene, addition of exogenous GG-PP (5 *μ*M) to Raw264.7 cells completely reversed the inhibitory effect of cerivastatin on LPS-induced IL-6 transcript levels (Figure 3A). In addition, GGTI 298, an inhibitor of geranylgeranyl transferase I, which transfers the geranylgeranyl moiety (from GG-PP) to proteins bearing a CaaX motif, dose-dependently (0–10 *μ*M) inhibited LPS-induced increase of IL-6 transcript levels in Raw264.7 cells (Figure 3B). Prenylated Rho family GTPases are known to regulate the organization of the actin cytoskeleton.^24^ In addition, macrophage elasticity and cellular shape, determined by the actin cytoskeleton, have been described as critical determinants of macrophage function, polarization, and inflammatory cytokine production.^25^ Based on our findings that statins inhibited IL-6 and TNF-*α* production in LPS-stimulated macrophages, we determined whether statins disrupt actin cytoskeleton organization in these cells. As seen in Figure 3C, exposure of LPS-stimulated Raw264.7 cells to cytochalasin D, an actin polymerization inhibitor, dose-dependently (0–5 *μ*M) decreased IL-6 mRNA levels. Moreover, LPS induced notable morphological changes in Raw264.7 cells, including elongation of cell shape and formation of filopodia. Treatment with cerivastatin completely inhibited cell elongation and filopodia formation (Figure 3D). Taken together, our data demonstrated that statins decreased IL-6 transcript levels in LPS-stimulated macrophages via reduction/inhibition of GG-PP and disruption of the actin cytoskeleton.

### LPS Stimulation of Macrophages Induces ADM Formation in Primary Pancreatic Acinar Cells Isolated From KC Mice: Inhibition by Statins

**Figure 4.**
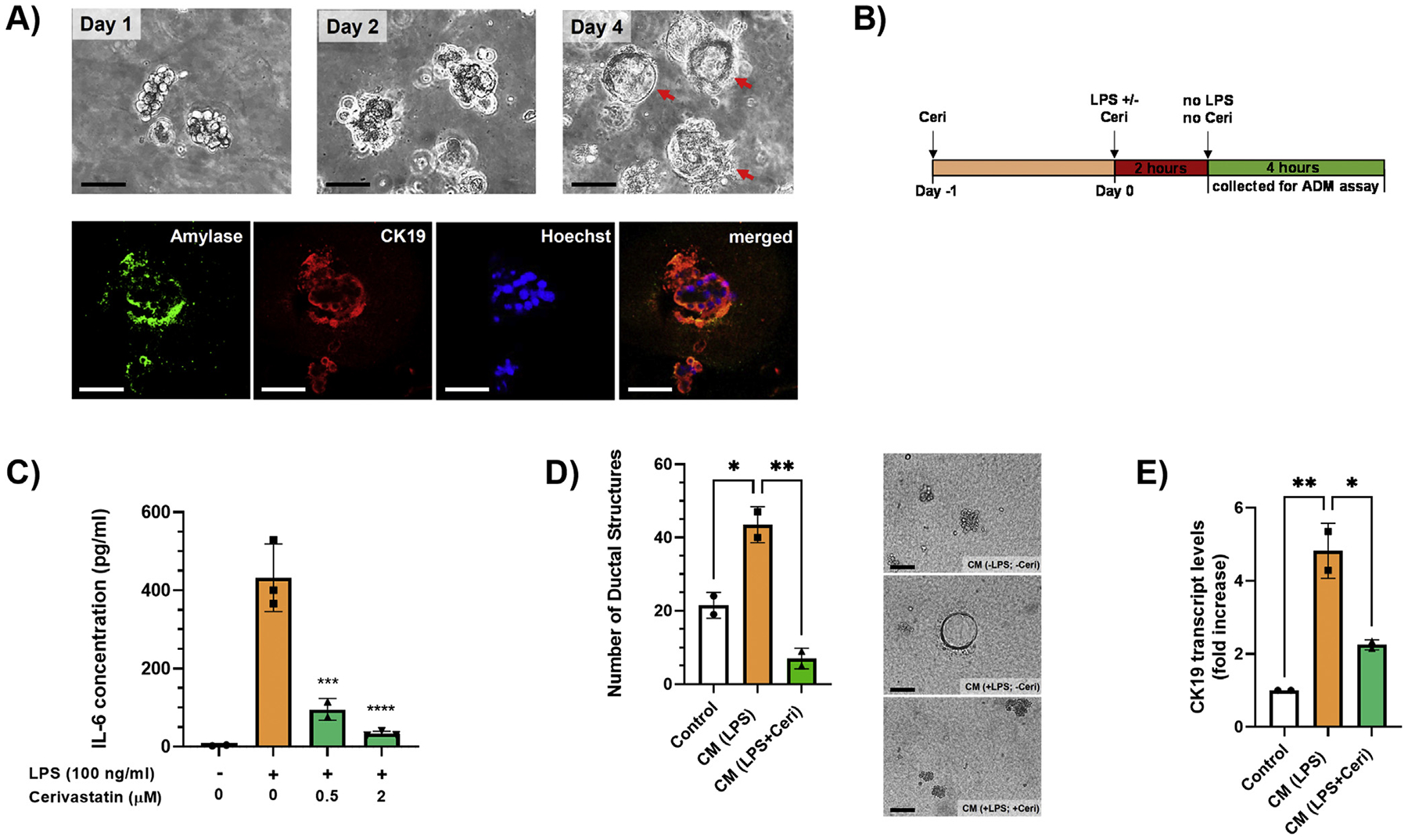
(A) Primary pancreatic acinar cells from KC mice were isolated and seeded in 3D culture in collagen. Representative structures were photographed under a brightfield microscope on days 1, 2, and 4 (upper row). Red arrows indicate ductal structures (ADM). Ductal structures at day 5 were stained with fluorescent antibodies against amylase (acinar cell marker; green) and CK19 (ductal cell marker; red) (lower row). Hoechst 33342 was used as a nuclear counterstain. Fluorescent images were merged using Photoshop. Scale bar, 100 *μ*m. (B) A schematic representation of the experimental design to collect the conditioned medium for the ADM assay. (C) IL-6 protein levels in the cell culture medium of the 4-hour collection were measured by ELISA. Data are presented as scatter plots with bars (mean ± SD). *P ≤ .05; ***P ≤ .001; ****P ≤ .0001. (D) Primary pancreatic acinar cells were isolated and seeded in 3D culture in collagen in 6-well plates. The conditioned cell culture media (CM) were added on top of the gel. Conditioned media were changed the next day (day 1) and then every 2 days. The total number of ducts per well were counted manually under a brightfield microscope on day 5 using 10× magnification. Data are presented as scatter plots with bars (mean ± SD) (left panel). *P ≤ .05; **P ≤ .01. Representative microscopic images are in the right panel. Scale bar, 100 *μ*m. (E) Cells from the ADM assay were extracted at day 5, RNA isolated, and CK19 mRNA levels were measured by real-time qPCR. Real-time PCR data were standardized to cells treated with control medium (set to 1). Data are presented as fold increase in scatter plots with bars (mean ± SD). *P ≤ .05; **P ≤ .01. ELISA, enzyme-linked immunesorbent assay; qPCR, quantitative polymerase chain reaction.

The preceding results prompted us to determine whether statins prevent ADM stimulated by inflammatory macrophages. As a first step, we confirmed ADM formation in primary acinar cells isolated from KC mice by microscopy and immunofluorescence. As shown in Figure 4A, acinar cell clusters were seen microscopically on day 1, while ductal structures were readily observed around day 4. Immunofluorescence staining demonstrated the simultaneous presence of acinar (amylase) and ductal (cytokeratin 19 [CK19]) cell markers in ductal structures, thereby confirming them as ADM lesions. These results are in agreement with a previous report showing that inflammatory macrophages stimulate ADM formation.^7^

To investigate whether the inhibition of inflammatory cytokines by lipophilic statins in macrophages is sufficient to counteract ADM formation, primary acinar cells from 3-month-old mice were exposed to the conditioned culture medium of Raw264.7 macrophages treated with control vehicle, LPS (100 ng/mL), or LPS (100 ng/mL) plus cerivastatin (0.5 *μ*M). To eliminate any potential direct effects on pancreatic acinar cells of remaining LPS and cerivastatin in the culture medium, Raw264.7 cells were stimulated with LPS in the absence or presence of cerivastatin for 2 hours. Then, the medium was replaced by fresh medium (not containing LPS/cerivastatin) for another 4 hours, which was subsequently used for the ADM formation assay (Figure 4B). In this 4-hour collection, IL-6 protein levels (as a representative of inflammatory cytokine production) were increased when Raw264.7 cells were stimulated by LPS prior, and this increase in IL-6 was prevented by cerivastatin (Figure 4C). The conditioned medium of LPS-treated Raw264.7 macrophages significantly increased ADM formation of primary pancreatic acinar cells after 5 days, which was completely prevented by cerivastatin (Figure 4D). However, IL-6 exogenously added to the 4-hour collection medium failed to reverse the effects of cerivastatin (Figure A2), suggesting that the overall inhibitory effect of statins on the expression of multiple cytokines in macrophages is likely responsible for the prevention of ADM formation. Quantitative PCR analysis from isolated ADM cells on day 5 demonstrated a significant increase of the ductal marker CK19 when acinar cells were exposed to the conditioned medium of LPS-treated macrophages, which again was decreased by cerivastatin (Figure 4E), thus confirming the data from the microscopic analysis. Taken together, these data clearly demonstrate that soluble factors released by inflammatory macrophages can stimulate the formation of ADM lesions and that exposure of macrophages to lipophilic statins can prevent that process.

### Oral Administration of Statins Decreases Intrapancreatic Macrophage Numbers, Inflammatory Cytokines, and Metaplastic/Neoplastic Lesions in the Conditional KrasG12D Mouse Model

**Figure 5.**
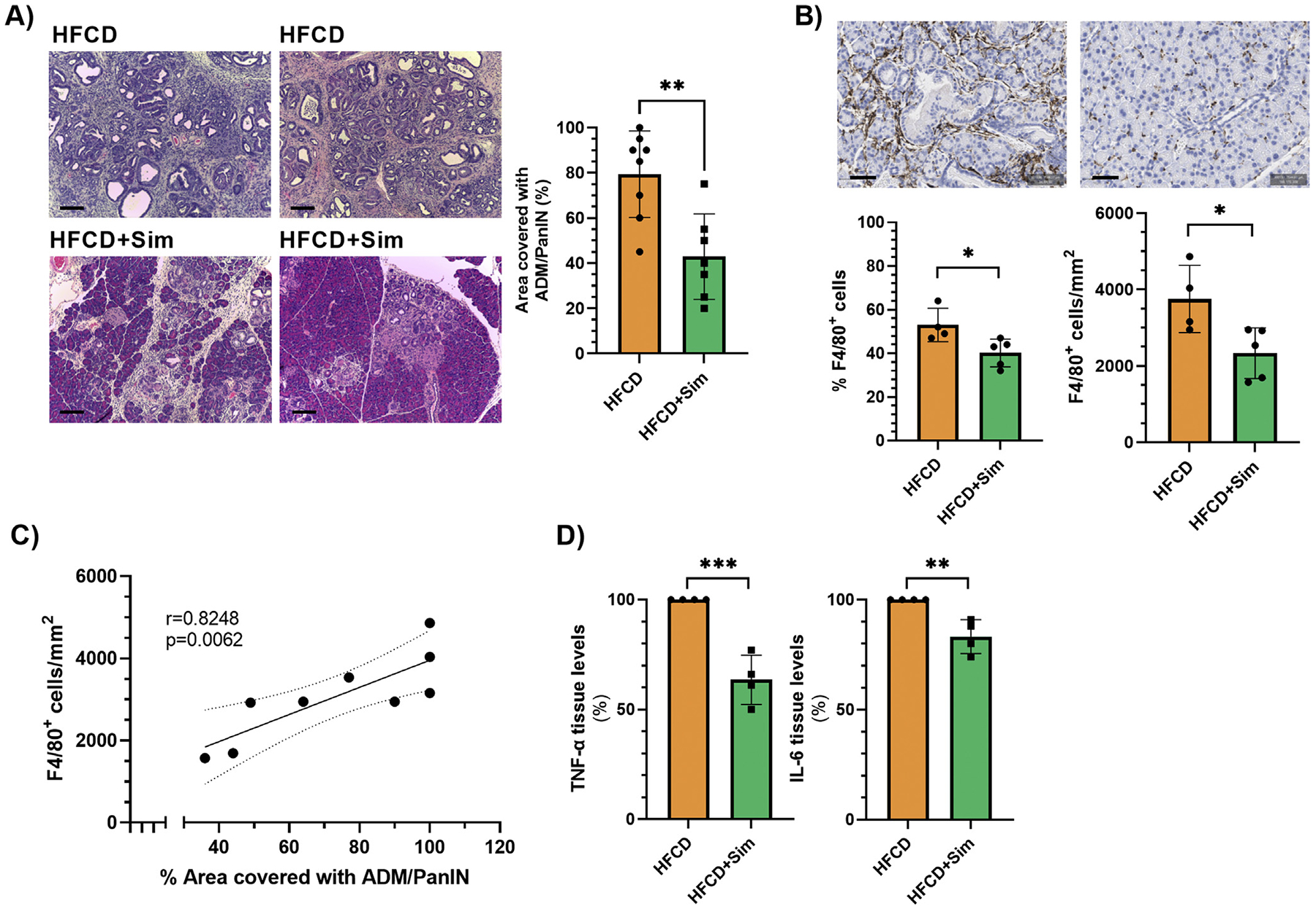
(A) Representative hematoxylin/eosin images from the pancreas of KC mice fed the high-fat, high-calorie diet (HFCD; upper row) or HFCD plus simvastatin (lower row). The area of the pancreas (in %; body and tail) covered in ADM/PanINs was quantified using QuPath (right panel; n = 8 for KC + HFCD and n = 7 for KC + HFCD + simvastatin). Scale bar, 100 *μ*m. Data are presented as scatter plots with bars (mean ± SD). **P ≤ .01. (B) Formalin-fixed, paraffin-embedded sections of the pancreas (body and tail) of KC mice fed HFCD (n = 4) or HFCD plus simvastatin (n = 5) were stained with antibodies against F4/80. Representative images in top row. Scale bar, 50 *μ*m. F4/80 positive staining was quantified using QuPath and presented as % F4/80 positive cells (in relation to all cells) (bottom left panel) or number of F4/80 positive cells per mm^2^ (bottom right panel). Data are presented as scatter plots with bars (mean ± SD). *P ≤ .05. (C) Correlation analysis with simple linear regression and 95% confidence interval of number of F4/80 positive cells per mm^2^ and % area covered in ADM/PanIN using Prim Version 9.1.2. r, Pearson correlation coefficient. (D) Protein levels of TNF-*α* (left panel) and IL-6 (right panel) in pancreatic lysates (n = 4 each) were measured by ELISA. Data were standardized to tissues from mice fed the HFCD (set to 100%). Data are presented as scatter plots with bars (mean ± SD). **P ≤ .01; ***P ≤ .001. ELISA, enzyme-linked immunosorbent assay.

After having demonstrated that lipophilic statins inhibit macrophage-stimulated ADM formation ex vivo, we then examined tissue samples from our previous animal study^20^ to determine the effects of simvastatin on intrapancreatic macrophage numbers, cytokine levels, and ADM formation. In that study, we have reported that oral administration of simvastatin for 3 months decreased the prevalence of PanIN-3 lesions in the KC mouse model with DIO (n = 14 each for KC + DIO and KC + DIO + simvastatin; female and male combined), which was histologically associated with reduced pancreatic inflammation.^20^ Further analysis of the pancreatic specimens in this current study revealed that oral administration of simvastatin decreased the formation of metaplastic/neoplastic ADM and PanIN lesions in KC mice. While ADM/PanIN lesions covered about 80% of the pancreas of KC mice fed the high-fat, high-calorie diet (HFCD) for 3 months, this percentage was decreased to about 43% in KC mice fed the obesogenic diet and simvastatin (*P* = .0026; Figure 5A). This was associated with reduced numbers of intrapancreatic F4/80 + macrophages as assessed by immunohistochemistry (Figure 5B). Oral administration of simvastatin reduced the percentage of F4/80 + cells (in relation to all nucleated cells) from 53% to 40% (*P* = .0285) as well as the number of F4/80 + cells/mm^2^ pancreas tissue (3752 vs 2334 cells/mm^2^; *P =* .0277) in KC mice fed the HFCD (Figure 5B). The number of intrapancreatic F4/80 + macrophages correlated thereby to the area of the pancreas covered in metaplastic/neoplastic lesions (r = 0.8248; *P* = .0062; Figure 5C). Furthermore, the pancreatic tissue of KC mice fed the HFCD and simvastatin had reduced levels of proinflammatory cytokines, including TNF-*α* and IL-6 (Figure 5D and Table A2). Immunofluorescence demonstrated that F4/80+;pSTAT1+ M1 macrophages were located within ADM lesions, while F4/80 +;YM-1 + M2 macrophages were predominantly found around PanINs (Figure A3), in agreement with a recent report.^26^ Together, these analyses suggest that the beneficial effects of simvastatin in attenuating PDAC development^20^ may at least in part be mediated by its effects on intrapancreatic macrophages, proinflammatory cytokine production, and ADM formation, thereby supporting our in vitro data.

## Discussion

Expression of oncogenic Kras, which is thought to be the initiating mutation in human PDAC, in murine pancreatic acinar cells leads to PDAC formation via the ADM-PanIN-PDAC sequence.^27^ The importance of M1-like macrophages and macrophage-secreted inflammatory cytokines in ADM formation has been demonstrated.^7,8^ Our previous study showing reduced acinar cell loss (as a marker of ADM formation) in KC mice with DIO and treated with simvastatin^20^ prompted us to speculate that the beneficial effects of statins were at least partially mediated by their inhibitory effects on macrophage-secreted inflammatory cytokines.

Our data clearly demonstrated that statins inhibit multiple LPS-stimulated inflammatory cytokines, for example, IL-1*α*, IL-1*β*, IL-6, TNF-*α*, and CCL5/Rantes. We selected IL-6 to investigate the underlying mechanisms of inhibitory statin action. This inhibitory effect in 2 murine macrophage/monocyte cell lines was limited to lipophilic statins, for example, cerivastatin, simvastatin, and fluvastatin, while the hydrophilic pravastatin failed to inhibit LPS-induced IL-6 production in macrophages. This may be explained by the lack of selective carrier-mediated transport system for pravastatin in these cell lines.^28^ The lack of effect of pravastatin (in contrast to lipophilic statins) has also previously been seen in PDAC cell lines.^20^ Our data are consistent with the known anti-inflammatory properties of statins^29^ and with reports describing a reduction of inflammatory cytokine production in macrophages.^30–32^ Mechanistically, we demonstrated that the inhibitory effects of statins on LPS-induced IL-6 production in macrophages are mediated by suppression of mevalonate synthesis. Mevalonate is a precursor for the formation of farnesyl pyrophosphate, 2 molecules of which are condensed to squalene by squalene synthase. Squalene is then converted through a multistep process to cholesterol. Our findings indicated that the inhibitory effect of statins on LPS-induced IL-6 production in macrophages is not mediated by the reduction of cholesterol. Alternatively, farnesyl pyrophosphate can also be converted to GG-PP. The transfer of the geranylgeranyl moiety to small Rho GTPases is important for its subcellular localization and function. In our studies, exogenously added GG-PP to macrophages reversed the inhibitory effects of statins on LPS-induced IL-6 production, suggesting that the mechanisms of statin action in macrophages involve inhibition of protein prenylation. Rho GTPases in turn regulate the actin cytoskeleton. It has recently been reported that cell shape, regulated by the actin cytoskeleton, modulates macrophage polarization and cytokine secretion.^25^ We showed that LPS stimulation induced cell elongation and filopodia formation in murine macrophages, which was inhibited by statins. Moreover, an actin polymerization inhibitor reduced LPS-increased IL-6 production in macrophages, thereby mirroring statin effects. The morphologic changes in LPS-stimulated macrophages elicited by statins may reflect a shift toward a M2-like macrophage phenotype.^33,34^ Together, our in vitro data provide evidence that the inhibitory effect of lipophilic statins on inflammatory cytokine production by macrophages involves the inhibition of mevalonate synthesis with a reduction in GG-PP and protein prenylation, leading to disruption of the actin cytoskeleton.

Furthermore, Rho GTPases and the actin cytoskeleton play an important role in regulating the subcellular localization and activity of Yes-associated protein (YAP) and WW domain containing transcription regulator 1 (TAZ), transcriptional coactivators in the Hippo pathway.^35^ Since statins are known inhibitors of YAP/TAZ in several cell types,^20,36^ it is conceivable that the inhibitory effect of lipophilic statins on inflammatory cytokine production by macrophages is mediated by inhibiting YAP and TAZ downstream of Rho GTPases and actin. This is supported by a recent study which shows that YAP mediates immune reprogramming in PDAC.^37^ In another report, YAP/TAZ expression is increased in inflammatory macrophages, and genetic deletion of YAP/TAZ decreased inflammatory cytokine production in macrophages.^38^ Furthermore, genetic deletion of both YAP and TAZ has been shown to inhibit pancreatic ADM formation in vivo.^39^ Finally, YAP and TAZ mediated the effects of statins in transformed breast epithelial cells.^36^

To test whether the effect of lipophilic statins on inflammatory cytokine production in macrophages translates into inhibition of ADM formation, we performed an ADM assay using primary pancreatic acinar cells and the conditioned medium of treated or untreated macrophages. We designed our ex vivo ADM assay specifically to eliminate any potential direct effect of statins on pancreatic acinar cells. Our data demonstrate that soluble factors from macrophages can elicit ADM formation, which was inhibited by prior exposure of macrophages to lipophilic statins. The addition of exogenous IL-6, however, failed to reverse the inhibitory effect of statins on ADM formation. This suggests that the inhibition of macrophage-elicited ADM formation by statins is mediated by multiple macrophage-secreted cytokines that are decreased by statins. This is supported by our RT2 Profiler Kit PCR Array, which detected a reduction in gene expression of multiple cytokines/chemokines in macrophages treated with statins. To support our data that statins inhibit ADM formation at least partially by inhibiting inflammatory cytokine production of macrophages, we further interrogated our tissue samples from a previous study, in which KC mice were treated with simvastatin.^20^ Our new findings showed that simvastatin administration was associated with reduced macrophage numbers and inflammatory cytokine levels in pancreatic tissue samples of KC mice. Interestingly, the number of intrapancreatic macrophages strongly correlated with the extent of ADM/PanIN lesions, further suggesting the importance of macrophages to PDAC development.

Taken together, our data provide evidence that the inhibitory effects of statins in LPS-induced IL-6 production in macrophages are mediated by the inhibition of mevalonate synthesis and the downstream pathway that involves GG-PP and the actin cytoskeleton but not cholesterol. Inhibition of macrophage-secreted inflammatory cytokines by statins may underlie the observed reduction of pancreatic ADM formation in KC mice. The effects of statins on macrophages may thereby collaborate with the direct inhibitory effects on transformed pancreatic epithelial cells,^20^ which cumulatively may reduce early PDAC development and progression. This notion is supported by epidemiologic and clinical studies that show beneficial effects of statins on lowering PDAC risk^11,13^ and improving the outcome of PDAC patients.^40–42^

## Methods

### Chemicals and Reagents

LPS was obtained from Invitrogen (Carlsbad, CA). Cerivastatin, simvastatin, fluvastatin, pravastatin, cytochalasin D, and squalene were from Sigma-Aldrich (St. Louis, MO). GG-PP, Ro 48-8071, and YM-53601 were purchased from Cayman Chemical (Ann Arbor, MI). GGTI 298 was purchased from R&D systems (Minneapolis, MN). Mevalonate was from Santa Cruz (Dallas, TX).

### Cells and Culture Conditions

Murine macrophage cell lines Raw264.6 and J774A.1 were obtained from the American Type Culture Collection (ATCC, Manassas, VA) and cultured in complete Dulbecco Modified Eagle Medium (DMEM) supplemented with 10% heat-inactivated fetal bovine serum (FBS) and 1% penicillin/streptomycin (100 U/mL) at 37 °C with 10% CO_2_.

### RNA Extraction and Quantitative RT-PCR

Total RNA was extracted from cells using the Tissue RNA Isolation Mini Kit (Biomiga, Burton, MI) according to the manufacturer’s instructions. Reverse transcription to generate cDNA was performed using the iScript Reverse Transcription Supermix (Bio-Rad, Hercules, CA). Quantitative PCR was performed by using the iTaq Universal SYBR Green Supermix (Bio-Rad). Relative mRNA concentration was normalized to the expression of 18s. Primer sequences were as follows: IL-6 forward, CTCTGCAAGAGACTTCCATCCA; IL-6 reverse, GACAGGTCTGTTGGGAGTGG; TNF-*α* forward, ACCCTCACACTCACAAACCA; TNF-*α* reverse, ACAAGGTACAACCCATCGGC.

### IL-6 and TNF-α Protein Levels

IL-6 and TNF-*α* protein levels in the cell culture supernatant were measured using the Mouse IL-6 and TNF-*α* enzyme-linked immunosorbent assay kits (RayBiotech, Norcross, GA), according to the manufacturer’s instructions.

### RT2 Profiler Kit PCR Array

RNA was extracted using the PureLink RNA Mini Kit (Thermo Fisher Scientific, Waltham, MA). Reverse transcription to generate cDNA was performed using the iScript Reverse Transcription Supermix (Bio-Rad). The Mouse Innate and Adaptive Immune Reponses RT2 Profiler Kit PCR Array (Qiagen, Germantown, MD) was used to detect 84 genes.

### Cholesterol Staining

Intracellular cholesterol was detected by the Cholesterol Cell-Based Detection Assay Kit (Cayman Chemical) according to manufacturer’s instructions. This assay is based on the interaction of filipin III with unesterified cholesterol, which alters the filipin fluorescence spectrum. Images were taken with a Nikon Eclipse 90i (Nikon, Tokyo, Japan) equipped with a 40× objective. Quantification of the intracellular cholesterol levels was accomplished using Image J software (version 1.51j8; National Institutes of Health, Bethesda, MD).

### Actin Staining

Raw264.7 cells were fixed with 3.7% methanol-free formaldehyde, and F-actin formation in Raw264.7 cells was observed after staining with Alexa Fluor 488 Phalloidin (Thermo Fisher Scientific) according to manufacturer’s instructions. Images were captured with an epifluorescence Zeiss (White Plains, NY) Axioskop and a Zeiss (Achroplan 40/.75W) objective.

### Primary Acinar Cell Isolation

Primary pancreatic acinar cells were isolated as published previously.^7^ Briefly, 1 mouse pancreas from 8- to 12-week-old C57BL/6J male mice was dissociated with collagenase (Sigma-Aldrich) at a concentration of 2 mg/mL in Krebs/Ringer solution with 0.25 mg/mL of trypsin inhibitor (Sigma-Aldrich) and 0.5-mM CaCl_2_ and 2% FBS (Thermo Fisher Scientific) with the pH adjusted to 7.3. Tissue was shaken at 37 °C at 225 rpm. The isolated pancreatic acinar cell suspension was filtered through a 100-*μ*M strainer, washed twice with Hanks’ Balanced Salt Solution, and allowed to incubate in DMEM media overnight for 24 hours in 6-well plates.

### ADM Assay

#### Preparation of Conditioned Cell Culture Media.

Two million Raw264.7 cells were seeded on 10-cm^2^ culture plates and incubated overnight in DMEM media (Thermo Fisher Scientific) without FBS. The media was replaced 24 hours later containing a control vehicle or 0.5-*μ*M cerivastatin for another 24 hours, followed by adding new cell culture medium with LPS (100 ng/mL), LPS (100 ng/mL) plus cerivastatin (0.5 *μ*M), or control vehicle. Two hours later, all cell culture media replaced with fresh serum-free DMEM media (no LPS, no cerivastatin). The enriched media was collected after 4 hours, filter-sterilized, and supplemented with 1% FBS, 2.5-*μ*M dexamethasone (Sigma-Aldrich), and 0.25-mg/mL trypsin inhibitor (Sigma-Aldrich).

#### ADM Assay.

The ADM assay was carried out as previously described^7^ with slight modifications. Briefly, dissociated primary pancreatic acinar cells were resuspended in DMEM with 1% FBS, 2.5 *μ*M of dexamethasone, and 0.25 mg/mL trypsin inhibitor. This suspension was mixed with a solution containing rat tail collagen (Thermo Fisher Scientific), 10× DMEM, and 0.34 M NaOH. The mixture was poured into 6-well plates, and the collagen gels were allowed to solidify for 30 minutes. Then the enriched cell culture media were added on top of the gel. In certain experiments, the enriched medium was spiked with exogenous IL-6 (PeproTech, Cranbury, NJ) at 2 or 12 ng/mL. The lower concentration of IL-6 was comparable to the concentration of IL-6 detected in the conditioned medium of RAW264.7 cells stimulated with LPS (100 ng/mL) for 6 hours. Enriched media were changed the next day (day 1) and then every 2 days. The total number of ducts per well were counted manually on day 5 using 10× magnification.

#### Immunofluorescence.

After duct formation (day 5), cells were fixed with 4% formaldehyde in phosphate-buffered saline, permeabilized with 0.5% Triton X-100, and blocked with 10% goat serum. Cells were then incubated with primary antibodies against amylase (Sigma-Aldrich) or CK19 (Leica Microsystems, Buffalo Grove, IL) at 4 °C overnight. Secondary antibodies (Invitrogen, Grand Island, NY) were added for 1 hour. Images were captured using a Nikon Eclipse 90i fluorescent microscope.

### Quantitative PCR of ADMs

At the end of the ADM assay (day 5), cells were extracted from the collagen gels as described.^7^ RNA was extracted according to manufacturer’s instructions with a PureLink RNA mini kit (Thermo Fisher Scientific). Reverse transcription to generate cDNA was performed using the iScript Reverse Transcription Supermix (Bio-Rad). Quantitative PCR was performed by using the iTaq Universal SYBR Green Supermix (Bio-Rad). Relative mRNA concentration was normalized to the expression of 18s. Mouse primer sequences were as follows: CK19 forward, CCTCCCGAGATTACAACCACT; CK19 reverse, GGCGAGATTGTCAATCTGT.

### ADM/PanIN Area

Sections of pancreatic tissue (body and tail; female and male mice combined) generated as described in a previous study^20^ were stained with hematoxylin and eosin, and the area covered by ADM and PanINs was quantified by QuPath (version 0.2.1.).^43^

### Immunohistochemistry and Immunofluorescence

Formalin-fixed, paraffin-embedded tissue sections (pancreatic body and tail; female and male mice combined) were dewaxed with xylene and rehydrated in an ethanol series. Antigen retrieval was performed in AR9 buffer (Akoya Biosciences, Menlo Park, CA), and sections were then immersed in blocking/diluent buffer (PerkinElmer, Richmond, CA). For immunohistochemistry, sections were incubated overnight with primary rat antimouse F4/80 antibody (#MCA497B; Bio-Rad) diluted 1:200 in blocking/diluent buffer. After washing, the Labelled Polymer-Dako REAL EnVision HRP kit (Agilent Dako, Santa Clara, CA) was then used to visualize antigen-antibody complexes.

For immunofluorescence, sections were incubated overnight with rat antimouse F4/80 (#MCA497B; Bio-Rad), rabbit antimouse pSTAT (Tyr701) (#44-376; Thermo Fisher Scientific), and rabbit antimouse Ym1 (#60130; STEMCELL Technologies, Cambridge, MA) antibodies (1:200 dilution in blocking buffer). Slides were then counterstained with multiplexing fluorescence kits from PerkinElmer: Opal 520 Reagent Pack, FITC, Opal 570 Reagent Pack, TRITC, and Opal 650 Reagent Pack, CY5, according to manufacturer’s instructions. 4′,6-diamidino-2-phenylindole was used for nuclear counterstain. Images were taken with a Nikon Eclipse 90i.

### Tissue IL-6 and TNF-α

Pancreas tissue homogenates from a previous study^20^ were profiled using the Mouse Cytokine Array C1000 (RayBiotech) following manufacturer’s instructions. The membrane-based proteomic array detects the relative levels of 96 cytokines and chemokines. Following the exposure to horseradish peroxidase, the membranes were imaged using the ChemiDoc Touch Imaging System (Bio-Rad). The signal intensity was normalized with positive controls and quantified with the ImageJ software (version 1.51j8).

### Statistical Analysis

Data are presented as mean ± SD. Differences in the mean of 2 samples were analyzed by an unpaired t test. Comparisons of more than 2 groups were made by a one-way ANOVA with post hoc Tukey analysis for pairwise comparisons and comparisons vs control. An *α* value of 0.05 was used to determine significant differences. Data were analyzed using GraphPad Prism 9.1.2 (San Diego, CA).

All authors had access to the study data and had reviewed and approved the final manuscript.

## Supplementary Material

2

1

3

4

5

## Abbreviations used in this paper:

ADM: acinar-to-ductal metaplasia
CK19: cytokeratin 19
DIO: diet-induced obesity
GG-PP: geranylgeranyl pyrophosphate
HFCD: high-fat high-calorie diet
IL-6: interleukin-6
KC: KrasG12D;p48-Cre
LPS: lipopolysaccharide
PanIN: pancreatic intraepithelial neoplasia
PDAC: pancreatic ductal adenocarcinoma
TAZ: WW domain containing transcription regulator 1
TNF-*α*: tumor necrosis factor-*α*
YAP: Yes-associated protein
