# Supplementary material for: Statins Inhibit Inflammatory Cytokine Production by Macrophages and Acinar-to-Ductal Metaplasia of Pancreatic Cells
Source: Gastro Hep Adv. 2022 Apr 25;1(4):640–51. doi: 10.1016/j.gastha.2022.04.012 (PMC9615480; doi:10.1016/j.gastha.2022.04.012)
Supplement: Table A1 [file mmc1.docx]

**Supplemental Table 1**

| **Gene** | **LPS**  Fold increase compared to control | **LPS + Cerivastatin**  Fold increase compared to control |
| --- | --- | --- |
| Apcs | 1.50 | 1.66 |
| C3 | 3.54 | 1.46 |
| C5ar1 | 1.75 | -1.41 |
| Casp1 | 2.23 | -1.18 |
| Ccl12 | 11.62 | 2.05 |
| Ccl5/RANTES | 79.21 | 16.02 |
| Ccr4 | 1.67 | 3.38 |
| Ccr5 | 2.83 | 7.92 |
| Ccr8 | 1.83 | 1.74 |
| Cd14 | 2.4 | 1.47 |
| Cd4 | 1.59 | 2.33 |
| Cd40 | 21.77 | 6.14 |
| Cd40lg | 3.21 | 4.14 |
| Cd80 | 3.20 | 1.45 |
| Cd86 | 13.6 | 5.77 |
| Crp | 1.05 | 1.03 |
| Cxcl10 | 2.71 | 1.94 |
| Cxcr3 | 1.48 | 1.43 |
| Ddx58 | 5.82 | 1.59 |
| Fasl | 1.39 | 1.38 |
| Foxp3 | 1.25 | 1.49 |
| Gata3 | 16.82 | 21.40 |
| H2-Q10 | 12.94 | 6.3 |
| Icam1 | -1.21 | -2.10 |
| Ifna2 | 26.89 | 49.64 |
| Ifnar1 | 1.10 | -1.89 |
| Ifnb1 | 30.41 | 59.92 |
| Ifng | 1.05 | -1.01 |
| Ifngr1 | 1.20 | 1.49 |
| Il10 | 3.36 | 3.66 |
| Il13 | 1.20 | -1.04 |
| Il17a | 2.18 | 4.85 |
| Il18 | 2.53 | 1.11 |
| Il1a | 424.74 | 177.94 |
| Il1b | 349.06 | 184.75 |
| Il1r1 | -1.28 | -1.05 |
| Il2 | 6.24 | 15.94 |
| Il23a | 3.78 | 5.71 |
| Il4 | 19.47 | 40.23 |
| Il5 | 7.52 | 14.88 |
| Il6 | 103.16 | 36.27 |
| Irak1 | -1.07 | -1.52 |
| Irf3 | 1.59 | 1.05 |
| Irf7 | 9.31 | 1.81 |
| Itgam | 3.78 | 2.11 |
| Jak2 | 4.42 | 2.57 |
| Ly96 | 2.11 | 1.08 |
| Lyz2 | -1.84 | -1.69 |
| Mapk1 | 1.81 | 1.13 |
| Mapk8 | 1.07 | -1.64 |
| Mpo | 1.83 | 3.41 |
| Mx1 | 2.38 | 2.26 |
| Myd88 | 1.58 | -1.43 |
| Nfkb1 | 3.05 | 1.63 |
| Nfkbia | -1.04 | 3.19 |
| Nlrp3 | 3.15 | 1.42 |
| Nod2 | 4.38 | 5.94 |
| Rag1 | 1.41 | 1.54 |
| Rorc | 5.34 | 8.37 |
| Slc11a1 | 4.61 | 1.94 |
| Stat1 | 4.15 | 1.12 |
| Stat3 | 2.51 | 1.07 |
| Stat4 | 4.18 | 11.09 |
| Stat6 | 1.77 | 1.21 |
| Tbx21 | 1.30 | 1.77 |
| Tlr1 | 4.81 | 5.50 |
| Tlr2 | -1.78 | -2.99 |
| Tlr3 | 3.18 | 1.00 |
| Tlr4 | 1.19 | -1.56 |
| Tlr5 | -1.03 | 1.07 |
| Tlr6 | 4.59 | 4.34 |
| Tlr7 | 1.62 | 1.30 |
| Tlr8 | 2.71 | 3.67 |
| Tlr9 | 2.09 | 3.13 |
| Tnf | 6.89 | 2.62 |
| B2m | 3.37 | 1.76 |

**Effect of cerivastatin on mRNA expression in macrophages.** RAW 264.7 cells were stimulated with LPS (100 ng/ml) for 6 hours without or with cerivastatin at 0.5 µM. Total RNA was extracted and mRNA levels of 84 genes were detected using the Mouse Innate and Adaptive Immune Reponses RT2 Profiler Kit PCR Array and real time qPCR. The fold increase in expression compared with RAW 264.7 macrophages without LPS and cerivastatin was calculated.
