# Supplementary material for: Statins Inhibit Inflammatory Cytokine Production by Macrophages and Acinar-to-Ductal Metaplasia of Pancreatic Cells
Source: Gastro Hep Adv. 2022 Apr 25;1(4):640–51. doi: 10.1016/j.gastha.2022.04.012 (PMC9615480; doi:10.1016/j.gastha.2022.04.012)
Supplement: Table A2 [file mmc2.docx]

**Supplemental Table 2**

| **Protein** | **%Expression (density)** |
| --- | --- |
| CCL20 | -69±23 |
| CXCL4 | -63±14 |
| VEGFR2 | -50±7 |
| CD62L | -48±17 |
| CCL5 | -42±22 |
| Resistin | -40±11 |
| LeptinR | -39±6 |
| TNFa | -37±11 |
| IL-3 | -35±20 |
| VEGF-D | -35±13 |
| VEGFR3 | -34±6 |
| MIP-1a | -31±18 |
| SCF | -29±11 |
| CCL25 | -26±15 |
| LIX | -25±8 |
| CXCL7 | -25±4 |
| CD106 | -25±7 |
| CCL11 | -25±10 |
| P-Selectin | -24±11 |
| IL-17A | -23±7 |
| CD54 | -22±8 |
| TPO | -21±10 |
| TNFRI | -21±3 |
| M-CSF | -20±7 |
| IL-10 | -19±4 |
| MIP-2 | -18±3 |
| IL-1a | -18±7 |
| CX3CL1 | -18±10 |
| IFNg | -17±8 |
| TNFRII | -16±9 |
| CRG-2 | -16±3 |
| IL-6 | -15±7 |
| CXCL13 | -14±4 |
| CD40 | -14±7 |
| GM-CSF | -14±3 |
| CCL27 | -13±10 |
| Shh-N | -13±9 |
| CXCL1 | -12±5 |
| CCL22 | -11±3 |
| IGFBP-2 | -11±9 |
| Leptin | -11±10 |
| CD26 | -11±4 |
| CD30 | -10±4 |
| IL-13 | -9±1 |
| IL-12 | -8±7 |
| VEGF-A | -7±2 |
| CCL24 | -6±10 |
| IL-3Rb | -6±8 |
| CD30 ligand | -6±11 |
| SSP1 | -6±14 |
| IL-9 | -6±4 |
| IL-2 | -5±6 |
| bFGF | -4±7 |
| IL-4 | -4±3 |
| I-309 | -4±4 |
| IGFBP-5 | -3±13 |
| IL-1b | -3±7 |
| MIP-1g | 0±11 |
| CXCL9 | 2±17 |
| CXCL16 | 3±8 |
| IGFBP-3 | 3±3 |
| CXCL15 | 3±14 |
| IL-12 p70 | 4±8 |
| XCL1 | 4±12 |
| TNFRSF18 | 5±6 |
| MCP-5 | 6±17 |
| GCSF | 10±5 |
| IL-5 | 12±3 |
| CCL17 | 14±19 |
| SDF-1a | 14±6 |
| IL-7 | 16±5 |
| Fc gamma RIIB | 17±21 |
| CXCL11 | 18±9 |
| MMP-2 | 21±5 |
| CCL2 | 21±4 |
| HGFR | 22±14 |
| TIMP-2 | 24±3 |
| TNFRSF11B | 27±11 |
| TNFRSF19 | 29±19 |
| IGF-2 | 30±13 |
| TNFSF11 | 31±9 |
| TSLP | 33±21 |
| Axl | 38±16 |
| IL-15 | 40±7 |
| Fit-3 Ligand | 44±25 |
| E-selectin | 45±15 |
| TNFSF6 | 47±21 |
| IL-17RB | 48±9 |
| IGFBP-6 | 50±23 |
| CCL19 | 65±14 |
| VEGFR1 | 71±24 |
| MMP3 | 76±10 |
| TIMP-1 | 77±11 |
| Dtk | 98±36 |
| Pro-MMP9 | 197±52 |
| IGF-1 | 312±85 |

**Pancreatic tissue levels of 96 cytokines and chemokines.** Pancreas tissue homogenates (n=4 for each KC+HFCD and KC+HFCD+Simvastatin) from a previous study^20^ were assayed using a Mouse Cytokine Array. The membrane-based proteomic array detects the relative levels of 96 cytokines and chemokines. The membranes were imaged and the signal intensity was normalized with positive controls and quantified.
